# Exploring endometriosis before surgical treatment: unraveling pain, sexual function and quality of life patterns

**DOI:** 10.61622/rbgo/2025rbgo47

**Published:** 2025-07-15

**Authors:** Juliana Olavo Pereira, Jaime Kulak

**Affiliations:** 1 Universidade Federal do Paraná Curitiba PR Brazil Universidade Federal do Paraná, Curitiba, PR, Brazil.

**Keywords:** Women's health, Endometriosis, Quality of life, Sexual health, Dyspareunia, Dysuria, Infertility, Surveys and questionnaires

## Abstract

**Objective:**

To identify pain, sexual function, and quality of life patterns in women with endometriosis, taking into consideration the American Society for Reproductive Medicine (ASRM) classification for endometriosis.

**Methods:**

A cross-sectional study of quantitative descriptive nature was conducted, including women with surgical recommendation due to endometriosis. The Numeric Pain Rating Scale, Endometriosis Health Profile, and Female Sexual Function Index tools were used for data collection. Descriptive and frequency analysis were employed. Using the K-means algorithm, cluster analysis was performed to group participants based on response similarities.

**Results:**

104 women with endometriosis were included with a median age of 35 years. The majority were classified as grade III (57.69%) and IV (25.96%) for endometriosis. There was a significant difference in the division of two clusters concerning ASRM, with ASRM IV women more frequently associated with Cluster B, while Cluster A being predominantly formed by ASRM III women. Cluster B showed significantly worse data for dyspareunia and dysuria pain levels and for all variables in the FSFI and EHP-30 instruments, except for infertility, which did not differ between the groups.

**Conclusion:**

ASRM classification is not directly related to clustering. Women diagnosed with endometriosis, mostly ASRM III and IV, exhibit two distinct patterns, with one group having worse pain, sexual function and quality of life scores compared to the other group. Infertility is a crucial aspect to study concerning the quality of life of women living with the disease and aspiring for motherhood irregardless of the clustering.

## Introduction

Endometriosis is a chronic gynecological disease that impacts women of reproductive age. It is defined by the presence of tissue resembling the endometrium located outside the uterine cavity, capable of reacting similarly to the endometrium during the menstrual cycle, thereby inducing a chronic inflammatory reaction.^(1)^

Approximately 6-10% of women of reproductive age are estimated to be affected by endometriosis,^(2)^ with 50% to 70% experiencing characteristic symptoms, even without a formal diagnosis of endometriosis.^(3)^ The most common complications of endometriosis include infertility and pelvic pain, such as dysmenorrhea, dyspareunia, chronic pelvic pain, dyschezia, and dysuria.^(4)^

The diagnosis of endometriosis is made through laparoscopic visualization and histological verification.^(5)^ The staging of the disease is based on these results, and one of the most commonly used instruments currently is the American Society for Reproductive Medicine classification (ASRM), which allows professionals to assess the severity and extent of endometriotic lesions.^(6)^ The classification does not consider pain, Sexual Function (SF), and Quality of Life (QoL) leading to approaches based on biological factors.^(7)^

However, the impact of endometriosis on the QoL and SF of affected women is considerable. Work, interpersonal relationships, sexuality, and family life can be compromised by the disease, and this negative impact can result in a reduction in QoL.^(8)^

The objective of this study was to identify pain, SF and QoL patterns in women with endometriosis, while considering the ASRM classification for endometriosis.

## Methods

This is a cross-sectional study of a quantitative and descriptive nature, developed in accordance with the Strengthening the Reporting of Observational Studies in Epidemiology (STROBE) guidelines.

All women with surgical recommendation due to endometriosis attending a tertiary health care service in Curitiba, Brazil, between February 20, 2021 and December 7, 2022, were invited to participate in the study. The Informed Consent Form (ICF) was presented to all of those who agreed to participate.

Women who were identified with clinical severe depression through the Beck Depression Inventory, using gonadotropin-releasing hormones (GnRH) or experiencing cognitive incapacity to respond to the designated questionnaire were excluded.

Data collection was done through self-directed questionnaires provided electronically via Google Forms. The sole components considered for the profile data of the study were: age, ethnicity and Body Mass Index (BMI)

The Beck Depression Inventory comprises 21 questions with scores ranging from 0 to 3, based on intensity. A validated Portugues version of the Beck Depression Inventory was used.^(9)^ The questionnaire categorization includes scores from 0 to 14 points (absence of depression), 15 to 19 points (mild depression), 20 to 29 points (moderate depression), and 30 to 63 points (severe depression).

Women were classified according to the ASRM classification based on surgical findings: stage I (minimal endometriosis) - score 1-5, isolated implants without significant adhesions; stage II (mild endometriosis) - score 6-15, superficial implants less than 5 cm, without significant adhesions; stage III (moderate endometriosis) - score 16-40, multiple implants with evident peritubal and periovarian adhesions; and stage IV (severe endometriosis) - score > 40, multiple superficial and deep implants, including endometriomas, dense and firm adhesions.^(10)^

The Numeric Pain Rating Scale (NPRS) was used to identify the pain level for dysmenorrhea, dysuria, dyspareunia, and dyschezia separately. It is a straightforward scale composed of numbers from 0 to 10, where participants choose the number that best represents the pain level for each situation.

To identify women's SF, the Female Sexual Function Index (FSFI^11^) was applied. The FSFI consists of 19 close-ended questions related to sexual activity within the 4 weeks prior to the examination and includes six domains: sexual desire, sexual arousal, lubrication, orgasm, satisfaction and pain. The scoring range varied from 0 to 5 or 1 to 5, depending on the question. Scores for each question were grouped by reference domain and multiplied by their corresponding factor. The overall score was the sum of all domain scores, where a lower score indicates worse SF. The cutoff point used to determine the risk for sexual dysfunction was 26.55.^(12)^

For the assessment of QoL, the Endometriosis Health Profile-30 (EHP-30)^(13)^ was applied. It is a specific questionnaire for women with endometriosis, consisting of 30 items that assess five dimensions: pain, control and powerlessness, emotional well-being, social support, and self-image. It also includes a modular questionnaire with 23 items distributed across six scales: sexual relationship, work, relationship with children, feelings about medical profession, treatment and infertility. Modules were answered exclusively by women who identified with - or were experiencing - the related topics. Each scale of the EHP-30 was transformed into a score from 0 to 100, where a lower score indicates better QoL.

Data was characterized considering quantitative variables: mean, standard deviation, minimum, maximum, median, and quartiles. For qualitative variables, only frequencies were considered.

The association between qualitative variables was assessed using Fisher's exact test.^(14)^ Additionally, the Mann-Whitney test^(14)^ was employed for comparing the qualitative explanatory variable with quantitative variables.

Cluster analysis using the K-means algorithm was conducted to group participants based on the results of scores from the tools utilized.^(15)^ Cluster analysis is an exploratory technique of multivariate data analysis that allows classification of a set of categories into homogeneous groups, observing only the similarities or differences between them.^(15)^ The scores used for analysis were from FSFI and the questions were from EHP-30.

The normality of variables was verified using the Shapiro-Wilk test,^(16)^ and a significance level of 5% was adopted. The analyses were conducted using the R computational language, version 4.1.1.^(17)^

This study followed the guidelines of Resolution 466/2012 from the Brazilian National Health Council, and was approved by Human Research Ethics Committee (4.450.209) of *Universidade Federal do Paraná* under the number *Certificado de Apresentação de Apreciação Ética (CAAE)* 39845920.3.0000.0102.

## Results

Participants included were women with surgical recommendation due to endometriosis, previously defined by Transvaginal Ultrasound with Intestinal Preparation, showing findings which were suggestive of deep endometriosis in retrocervical, vaginal, intestinal, bladder, or ureteral regions, or endometrioma, along with the presence of suggestive symptoms such as dysmenorrhea, chronic pelvic pain, dysuria, dyschezia and infertility. The initial study cohort was comprised of 109 women. However, five of them were excluded based on their depression scores, resulting in a total of 104 participants. Data revealed that the majority of women identified themselves as white (76.92%, n=80), followed by mixed race (20.19%, n=21), black (1.92%, n=2), and of Asian heritage (0.96%, n=1). The median age of the participants was 35 [32-40] years, with a minimum age of 21 and a maximum of 45 years old. The median BMI was 26,02 [IIQ 22,95-28,96], ranging from a minimum of 17.12 to a maximum of 41.27 kg/m². Regarding the ASRM classification, 10 women (9.62%) were classified as I, 7 (6.73%) as II, 60 (57.96%) as III, and 27 (25.96%) as IV. A risk for sexual dysfunction was identified in 72 women. Descriptive data for continuous variables of pain, FSFI and EHP-30 are presented in table 1. The highest median for pain was recorded in dysmenorrhea, followed by dyspareunia. The median overall score for FSFI was 22.3, with pain, desire and arousal showing the worse individual scores. In EHP-30, the median overall score was 46.25, with the Control and Powerlessness dimension and the Infertility module presenting the highest individual scores, indicating worse QoL.

**Table 1:** Descriptive table of pain, FSFI and EHP-30 continuous variables

Variable (n)		Median [IQR]	Min – Max
Pain			
	Dyspareunia (89)	4 [1-6]	0 – 10
	Dysuria (104)	0 [0-4]	0 – 10
	Dyschezia (104)	2 [0-6]	0 – 10
	Dysmenorrhea (104)	7 [2-10]	0 – 10
FSFI			
	Desire (104)	3.6 [2.4-4.2]	1.2 – 6
	Arousal (104)	3.6 [2.4-4.8]	0 – 6
	Lubrication (104)	4.5 [2.62-5.4]	0 – 6
	Orgasm (104)	4 [1.9-4.9]	0 – 6
	Satisfaction (104)	4 [2.8-5.2]	0,8 – 6
	Pain (104)	3.6 [1.2-4.8]	0 – 6
Total (104)		22.3 [16.03-28.12]	2 – 36
EHP-30			
	Pain (104)	39.77 [6.25-52.84]	0 – 100
	Control and powerlessness (104)	52.08 [12.5-75]	0 – 100
	Emotional well-being (104)	47.92 [28.12-62.5]	0 – 100
	Social support (104)	50 [23.44-70.31]	0 – 100
	Self-image (104)	50 [16.67-68.75]	0 – 100
Total (104)		46.25 [21.67-61.04]	0 – 100
Modules EHP-30^b^			
	1 - Work (83)	20 [5-57.5]	0 – 85
	2 - Relationships with children (36)	50 [25-75]	0 – 100
	3 - Sexual relationships (99)	45 [20-72.5]	0 – 100
	4 - Medical profession (104)	0 [0-1.56]	0 – 93.75
	5 - Treatment (103)	16.67 [0-50]	0 – 100
	6 - Infertility (79)	62.5 [37.5-81.25]	0 – 100

IQR: Interquartile range (1st and 3rd quartile). a. Variables do not have a normal distribution.

b Modules answered only by women who understood the situation presented as important

**Figure 1:**
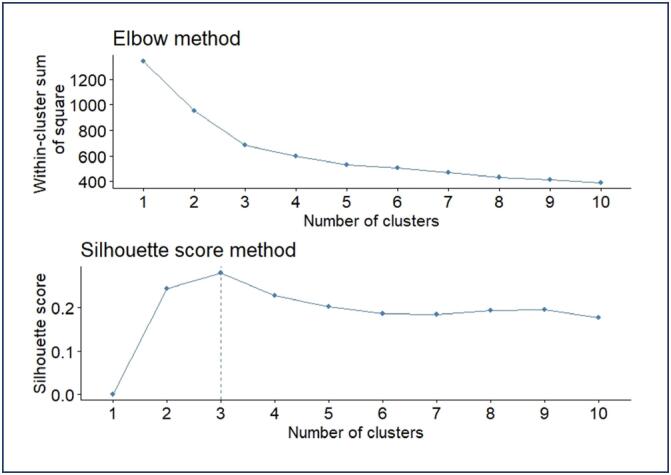
Graphs of selection of number of clusters

The data underwent cluster analysis using the K-means algorithm. To assist in determining the number of clusters, graphs from the Elbow and average silhouette methods were utilized, both of which indicated the point three, as shown in figure 1. In this case, data from the FSFI and EHP-30 were tested for the formation of two, three and four groups.

**Figure 2:**
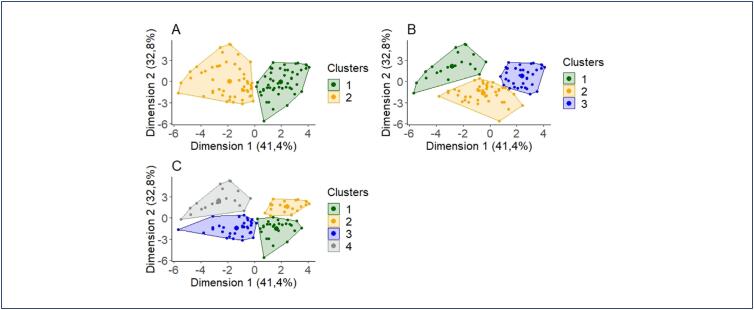
Graph of calculated clusters

Subsequently, a grid of K values (clusters) was tested, taking into consideration the number of groups indicated earlier by the graphs (Figure 2).

The clusters determined by the K-means algorithm were compared with the results of the ASRM classification (Table 2), showing that only the classification into two groups was significant (p-value=0.014) for the ASRM classification. Therefore, it was decided to use two groups to analyze the relevant quantitative and qualitative variables.

**Table 2:** Cross-tabulations between clusters and ASRM classification

Number of groups	Cluster	I	II	III	IV	p-value
		n(%)	n(%)	n(%)	n(%)	
2	Group A	4(40)	3(42.86)	37(61.67)	7(25.93)	0.014
	Group B	6(60)	4(57.14)	23(38.33)	20(74.07)	
3	Group A	2(20)	2(28.57)	11(18.33)	7(25.93)	0.422
	Group B	5(50)	3(42.86)	23(38.33)	15(55.56)	
	Group C	3(30)	2(28.57)	26(43.33)	5(18.52)	
4	Group A	2(7.41)	2(10)	2(8.7)	4(11.76)	0.245
	Group B	1(3.7)	2(10)	2(8.7)	2(5.88)	
	Group C	20(74.07)	10(50)	16(69.57)	14(41.18)	
	Group D	4(14.81)	6(30)	3(13.04)	14(41.18)	

Fisher's test (significance level of 5%)

To investigate the origin of the significant difference in the ASRM distribution, a multiple comparison analysis was conducted, as indicated in table 3. The significant disparity was evident only when comparing ASRM classifications III and IV with each other. Cluster A includes a higher prevalence of ASRM III patients, while 74.07% of women classified as ASRM IV are allocated to Cluster B.

**Table 3:** Cross-tabulations between qualitative variables and the grouping of two clusters

Variables	ASRM n(%)	Cluster A n(%)	Cluster B	p-value
	n(%)	n(%)		
ASRM Classification	I (10)	4(7.84)	6(11.32)	0.014
	II (7)	3(5.88)	4(7.55)	
	III (60)	37(72.55)	23(43.4)	
	IV (27)	7(13.73)	20(37.74)	
ASRM Classification - Multiple Comparisons^a^				
II *Versus* I				0.999
III *Versus* I				0.530
IV *Versus* I				0.530
III *Versus* II				0.530
IV *Versus* II				0.530
IV *Versus* III				0.015

Fisher's test (significance level of 5%).

a Bonferroni multiple comparison test

The quantitative variables compared between the two clusters are presented in table 4. Age and BMI did not show significant differences, indicating homogeneity between the two clusters in these aspects. Regarding the pain level, there were no significant differences for dysmenorrhea and dyschezia, although cluster B exhibited worse outcomes.

**Table 4:** Cross-tabulations between quantitative variables and the grouping of two clusters

Variables (n)	ASRM (n)	Median [IQR]	Min. - Max.	p-value
Profile				
Age (104)	A (51)	35 [32 - 39]	21 – 44	0.599
	B (53)	35 [32 - 41]	23 – 45	
BMI (104)	A (51)	25.39 [23.08 – 28.83]	17.12 – 41.27	0.730
	B (53)	26.23 [22.95 – 29.03]	19.95 – 37.47	
Pain				
Dyspareunia (89)	A (48)	*3 [1 - 5]*	0 - 10	0.012
	B (41)	*5 [3 - 7]*	0 - 10	
Dysuria (104)	A (51)	*0 [0 - 2]*	0 - 10	0.008
	B (53)	*2 [0 - 5]*	0 - 10	
Dyschezia (104)	A (51)	*1 [0 – 4.5]*	0 - 10	0.076
	B (53)	*3 [0 - 6]*	0 - 10	
Dysmenorrhea (104)	A (51)	*6 [1 - 9]*	0 - 10	0.100
	B (53)	*8 [4 - 10]*	0 - 10	
FSFI				
Desire (104)	A (51)	3.6 [3.6 – 4.8]	2.4 – 6	<0.001
	B (53)	2.4 [1.8 – 3.6]	1.2 – 5.4	
Arousal (104)	A (51)	4.8 [3.9 – 5.1]	2.7 – 6	<0.001
	B (53)	2.4 [0 – 3.6]	0 – 4.5	
Lubrication (104)	A (51)	5.4 [4.8 - 6]	2.1 – 6	<0.001
	B (53)	3 [0 – 3.9]	0 – 6	
Orgasm (104)	A (51)	5.2 [4.4 – 5.6]	2.4 – 6	<0.001
	B (53)	2 [0 – 3.6]	0 – 4.8	
Satisfaction (104)	A (51)	5.2 [4.6 - 6]	1.2 – 6	<0.001
	B (53)	3.2 [2.4 - 4]	0.8 – 5.6	
Pain (104)	A (51)	4.8 [3.6 - 6]	0 – 6	<0.001
	B (53)	1.6 [0 – 4.4]	0 – 6	
Total (104)	A (51)	28.2 [25.2 – 30.45]	21 – 36	<0.001
	B (53)	16.3 [7.6 – 20.6]	2 – 26.2	
EHP-30				
Pain (104)	A (51)	20.45 [0 – 44.32]	0 – 100	<0.001
	B (53)	50 [34.09 – 72.73]	0 – 100	
Control and Powerlessness (104)	A (51)	20.83 [2.08 – 60.42]	0 – 100	<0.001
	B (53)	66.67 [41.67 – 83.33]	0 – 100	
Emotional Well-being (104)	A (51)	45.83 [25 - 50]	0 – 100	0.003
	B (53)	54.17 [37.5 – 70.83]	0 – 91.67	
Social Support (104)	A (51)	37.5 [12.5 – 62.5]	0 – 100	0.004
	B (53)	62.5 [31.25 – 81.25]	0 – 93.75	
Self-image (104)	A (51)	41.67 [12.5 – 54.17]	0 – 100	0.003
	B (53)	58.33 [41.67 – 83.33]	0 – 100	
Total (104)	A (51)	31.67 [14.58 – 47.08]	0 – 100	<0.001
	B (53)	55 [45.83 – 69.17]	0 – 92.5	
Modules EHP-30				
1 – Work (83)^a^	A (40)	15 [0 - 25]	0 – 80	0.010
	B (43)	40 [10 – 62.5]	0 – 85	
2 – Relationship with Children (36)^a^	A (17)	37.5 [12.5 - 50]	0 – 75	0.0117
	B (19)	62.5 [50 - 75]	0 – 100	
3 - Sexual Relationships (99)^a^	A (47)	30 [12.5 – 47.5]	0 – 80	<0.001
	B (52)	65 [45 - 75]	0 – 100	
4 - Medical profession (104)^a^	A (51)	0 [0 - 0]	0 – 68.75	0.009
	B (53)	0 [0 – 12.5]	0 – 93.75	
5 - Treatment (103)^a^	A (50)	16.67 [0 - 33.33]	0 – 100	0.036
	B (53)	41.67 [8.33 – 58.33]	0 – 100	
6 - Infertility (79)^a^	A (42)	68.75 [43.75 – 81.25]	0 – 100	0.996
	B (37)	62.5 [37.5 – 81.25]	0 – 100	

Mann-Whitney test (significance level of 5%). IQR: Interquartile Range (1st and 3rd quartile).

a Modules answered only by women who identified with the situation presented

The results show that Cluster A, composed of the larger portion of women with ASRM III, presents significantly better median [IQR] values for dyspareunia, dysuria, FSFI and EHP-30, with the exception of the Infertility module, when compared to women in Cluster B, formed by the larger portion of women classified with ASRM IV. The overall scores of FSFI and EHP-30 illustrate the significant difference found between the clusters. The median total FSFI score for Cluster A was 28.2 with a minimum of 21 and a maximum of 36, while for Cluster B, there is a drastic drop in these values, with a median of 16.3 points, a minimum of 2, and a maximum of 26.2. In the overall score of EHP-30, the median for Cluster A was 31.67 [IQR 14.58 - 47.08], while Cluster B had a median of 55 [IQR 45.83 - 69.17]. The comparison between the two clusters regarding the risk for sexual dysfunction is presented in table 5. The results show that cluster B presented significant worse data on sexual dysfunction compared to cluster A. Cluster B consisted of 100% of women with risk for sexual dysfunction, whereas the number of women with dysfunction in cluster A was only 37.25%.

**Table 5:** Cross-tabulations between sexual dysfunction and the grouping of two clusters

Variables	Cutoff	Cluster A	Cluster B	p-value
		n(%)	n(%)	
Sexual dysfunction	<=26.55(10)	19 (37.25)	53 (100)	<0.001
	>26.55 (7)	32 (62.75)	0(0)	

Chi-square test (significance level of 5%)

## Discussion

Endometriosis is a chronic disease with significant social, economic and public health implications. The negative experiences endured by women can influence SF and QoL. Pain, fatigue, depression, anxiety and infertility are the main influencing factors.^(18)^ The aim of this study was to identify pain, SF and QoL patterns in women with endometriosis before the surgical treatment, taking into consideration the ASRM classification.

The collected data show that the majority of women had more severe lesions (83.65%), classified as stage III and IV for endometriosis, which means that the data presented cannot be extrapolated to all women diagnosed with endometriosis. Our participants were more likely to be women with refractory endometriosis to clinical treatment or with severe symptoms. Overall, it is more common for women in pre or postoperative phases to have advanced stages of endometriosis.^(19,20)^ These findings support the recommendation for surgical intervention, as the procedure is advised based on the severity of lesions, failure of clinical treatment and infertility.^(21)^

The primary symptoms of endometriosis are chronic pain and infertility. Nevertheless, the infertility module was the variable that showed greater similarity among women, regardless of the clusters. The Infertility module in the EHP-30 was designed to gauge the impact of the potential inability to have children on women's lives. In general, infertility or the prospect of it can evoke the feeling of frustration and fear in couples aspiring to have children.^(22)^

The relationship between endometriosis and infertility has been extensively investigated. Around 30-50% of women diagnosed with endometriosis are infertile, presenting a four times increased risk of infertility compared to healthy women. Even though it is known that mechanical factors, pain and modifications of gamete functions and transport are involved, infertility in women with endometriosis has not been yet completely understood.^(23)^

The pain level concerning dysmenorrhea and dyschezia also did not show a significant difference between clusters A and B, although women in cluster B exhibited worse scores. Nevertheless, when pain was assessed in the context of QoL and SF, both exhibited notable distinctions, with poorer scores associated with cluster B. Pain is one of the most common symptoms in women with endometriosis, being a crucial parameter in clinical practice.

With the exception of pain and infertility, all other variables presented in the study significantly differed between clusters A and B. Women diagnosed with endometriosis in this study clearly exhibited two distinct behavior patterns, with worse scores attributed to cluster B. Although the cluster division is not dependent on ASRM, it is evident that women with significantly better scores are predominantly found in Cluster A, which includes a higher proportion of women with ASRM III. Conversely, Cluster B shows worse scores overall, with 74.07% of ASRM IV women allocated to this cluster.

The reasons for the higher frequency of ASRM III women in Group A and ASRM IV women in Group B could not be fully understood in this study. However, in practical terms, there is a very thin line between women with ASRM III and IV, and they may sometimes be misclassified. Nevertheless, it is concluded that the ASRM classification cannot predict the intensity of pain and patterns of SF and QoL in women living with the disease.

The ASRM classification is crucial and widely used in clinical settings to identify the severity of lesions caused by endometriosis and assist in treatment selection. Despite its importance, there is still a considerable amount of work to be done regarding the classification of this complex and challenging disease.^(6)^

This study was limited to a low number of women ASRM I and II, which may have influenced the final findings. New studies that include an equivalent number of women among different classifications, especially I and II, may find different results than the present study. Additionally, the inclusion of different variables may aid in a better understanding of these patterns.

SF variables and dyspareunia, which are directly related to sexual health, showed significantly worse function in cluster B. When considering the cutoff values for sexual dysfunction (<26.55),^(12)^ 100% of women in cluster B had risk for sexual dysfunction, while only around 37% in cluster A.

It is estimated that two-thirds of women with endometriosis have some form of sexual dysfunction related to the disease.^(24)^ Dyspareunia has been considered one of the main negative influencers of sexual health in women with endometriosis, being one of the reasons for refusal and interruption of sexual activity.^(25,26)^ Maintaining regular sexual activity while experiencing dyspareunia can be a challenge for women and their partners, but adaptations can be made to maintain a healthy sexual life.^(25)^

## Conclusion

The data presented show that women with surgical indication are more frequently classified as ASRM III and IV, resulting in the high number of women with this classification in our study. This means that the results found more accurately represent the reality of women living with more severe cases of endometriosis, failure in clinical treatment or infertility. For women in these conditions, the data reinforces that endometriosis interferes with the QoL and SF. Two distinctly different behaviors among them were found using the questionnaires, with one group showing worse scores in all tested variables. This means that there is a group of women with endometriosis who present, at the same time, more severe dyspareunia, risk for sexual dysfunction and impaired QoL. While another group, with similar characteristics tested in the study, presents a better pattern in all these aspects. Infertility emerged as an important point in the study, negatively affecting the QoL of women who wish to have children regardless of the cluster. The fact that there was no difference between the groups reinforces the negative impact of infertility, or the fear of it, in the lives of women with endometriosis. Studies aiming to understand the SF and QoL patterns of women with endometriosis can be useful to identify their individual needs. There is clearly a division of behavior among women diagnosed with endometriosis, and a better understanding of the factors determining this behavior can assist in clinical practice.
